# Apoptin-modified human mesenchymal stem cells inhibit growth of lung carcinoma in nude mice

**DOI:** 10.3892/mmr.2015.3501

**Published:** 2015-03-17

**Authors:** JINGCHUN DU, YANLING ZHANG, CHUN XU, XIA XU

**Affiliations:** 1 Department of Laboratory Medicine, First Affiliated Hospital, Guangzhou Medical University, Guangzhou, Guangdong 510120, P.R. China; 2 Department of Clinical Immunology, School of Kingmed Diagnostics, Guangzhou Medical University, Guangzhou, Guangdong 510182, P.R. China; 3 Department of Laboratory Medicine, Fifth Affiliated Hospital, Guangzhou Medical University, Guangzhou, Guangdong 510700, P.R. China

**Keywords:** mesenchymal stem cells, apoptin, lentivirus, lung cancer

## Abstract

Human mesenchymal stem cells (MSCs) represent a novel carrier for gene therapy and apoptin is a potential tumor-selective apoptosis-inducing protein. In the present study, the anti-tumoral effect of MSCs modified with apoptin against lung carcinoma was evaluated. Apoptin protein was expressed in a prokaryotic expression system and purified by affinity chromatography. Subsequently, anti-apoptin antibody was prepared by immunizing BALB/c mice with purified apoptin protein. Human MSCs were isolated, amplified and transduced with lentiviral vectors encoding full-length apoptin, in which the secretory signal and protein transduction sequence were added into the amino terminus to assist apoptin in entering into target cells. The differentiation and apoptin expression of apoptin-modified MSCs were confirmed. Subsequently, the anti-tumor effect of apoptin-modified MSCs was measured *in vitro* and *in vivo*. Following modification with apoptin, MSCs retained their differentiation capacity, and successfully synthesized and secreted apoptin, which entered target cells and selectively induced lung cancer cell apoptosis through activating caspase-3. The percentage of tumor cells with activated caspase-3 in the apoptin-modified MSCs group was markedly higher than that in the MSCs group (16.5±2.9% at 24 h and 27.3±2.0% at 48 h vs. 3.4±1.1% at 24 h and 2.2±0.6% at 48 h). When injected into nude mice, apoptin-modified MSCs inhibited the growth of lung carcinoma compared with that in the control groups (0.14±0.02 g vs. 0.21±0.04 g vs. 0.31±0.05 g, P<0.05). The results of the present study provided preclinical support of apoptin-based cancer therapy with MSCs as cellular vehicles.

## Introduction

The potential of gene therapy as a treatment of cancer has been supported by preclinical and clinical experiments (1,2). However, the detailed approach, such as guidelines for the selection of suitable gene delivery vehicles and therapeutic genes, remains to be further developed. As mesoderm-derived stem cells, mesenchymal stem cells (MSCs) are easily obtained from different tissues, including bone marrow, adipose tissue and umbilical cord (3,4). Following infusion into the body, MSCs were shown not to cause host immunological rejection compared with oncolytic viruses and were able to selectively migrate into tumor tissue (5–8). Due to their low immunogenicity and specific tropism toward tumors, MSCs have been used as potent cellular vehicles to treat tumors following being engineered with different anti-cancer agents (9,10).

Apoptin is a chicken anemia virus-derived non-structural protein and composed of 121 amino acids, which induces apoptosis in a broad range of transformed and cancerous cells, but not in non-transformed and primary cells (11,12). Although the mechanisms of cell death induced by apoptin remain to be elucidated, previous studies have demonstrated that apoptin may inhibit tumor growth *in vivo* (13,14). In these studies, apoptin was delivered as a nucleotide by virus carriers or directly injected into the body as a recombinant protein. However, these administration routes may cause the recipient to undergo a rejection reaction or may not reach effective concentration due to a short half-life and the limitation of the maximum tolerated dose (7,15).

Based on this background, it was the aim of the present study to assess whether MSCs could be modified with apoptin to inhibit tumor growth. In the present study, it was first demonstrated that MSCs could be efficiently modified with apoptin using a lentivirus system and delivery of apoptin could induce apoptosis of lung cancer cells through activating caspase 3. *In vivo* models further confirmed the anti-tumor effects of MSCs modified with apoptin.

## Materials and methods

### Culture and preparation of human MSCs and other cell lines

The present study was approved by the ethics committee of the First Affiliated Hospital of Guangzhou Medical University (Guangzhou, China). Human bone marrow-derived MSCs were isolated, expanded and induced to differentiate as previously described (16). In the current study, the bone marrow samples were derived from two male volunteers, who were 26 and 35 years old, respectively. The individuals had been admitted to hospital due to a road traffic accident. The bone marrow was collected between May 2012 and January 2013. Informed consent was provided by all individuals. The separated MSCs were sub-cultured at a concentration of 1×10^4^ cells/cm^2^ in low-glucose Dulbecco’s modified Eagle’s medium with 10% fetal bovine serum and were used for experiments at passages 4–8. The human lung cancer cell lines H460 and H1299 (American Type Tissue Collection, Rockville, MD, USA), and normal fibroblast cells were cultured in RPMI 1640 media (HyClone Laboratories, Inc., Logan, UT, USA) supplemented with 10% fetal bovine serum and modified with humanized Renilla green fluorescence protein (hrGFP; Invitrogen Life Technologies, Carlsbad, CA, USA) as previously described (16). Subsequently, the cell lines were termed H460 hrGFP, H1299 hrGFP and Fibroblast hrGFP, respectively.

### Construction of vectors

To prepare prokaryotic expression vector pET28b-apoptin, an apoptin sequence derived from multiplex polymerase chain reaction (PCR) was first amplified by PCR using primer 1, 5′-CATGCCATGGTAAACGCTCTCCAAGAAG-3′ and primer 2, 5′-AAATATGCGGCCGCCAGTCTTATACACC-3′ (Invitrogen Life Technologies). Subsequently, the PCR products were digested with *Nco*I and *Not*I, gel-purified and then ligated into vector pET28b prepared in the same manner. The ligation product was transformed into DH5α and recombinant clones were selected. PCR, double enzyme cutting and sequencing were used to further confirm the recombinant vector pET28b-apoptin.

To prepare the lentiviral expression vector, pLV/Final-puro-EF1α-apoptin, the apoptin sequence carrying the secretory signal and the protein transduction domain (PTD) signal was cloned into pDONR^TM^221 (Invitrogen Life Technologies) using the BP recombination reaction to generate entry clone pDown-apoptin. Subsequently, pUp-EF1α and pDown-apoptin were recombined into pDest using the LR recombination reaction to construct pLV/Final-puro-EF1α-apoptin according to the manufacturer’s instructions (Invitrogen Life Technologies). Finally, PCR and sequencing were used to identify the correction of the recombinant eukaryotic expression vector.

### Preparation of anti-apoptin antibody

The recombinant pET28b-apoptin vector was constructed in our laboratory and transformed into *E.coli* BL21 (DE3; Fulengen Inc., Guangzhou, China). A positive clone was induced to express target protein using isopropyl β-D-1-thiogalactopyranoside (IPTG; 0.1 mM) and relatively low temperature (26°C). Following sonication and centrifugation at 10,000 × g for 30 min, cell pellets were resolved with phosphate-buffered saline (PBS) containing urea (8 M), applied to a Ni^2+^-chelating column (GE Healthcare, Beijing, China), then eluted using a stepwise gradient of PBS containing urea (8 M) and different concentrations of imidazole (from 20 to 400 mM). The eluates were collected and identified using SDS-PAGE analysis. The fraction containing the recombinant protein were dialyzed with PBS buffer, concentrated using a concentrator plus (Eppendorf, Hamburg, Germany) and stored at −20°C for future use. A total of four five-week-old male BALB/c mice were supplied by the Experimental Animal Center of Guangdong Province (Foshan, China). The mice were housed with *ad libitum* access to food and water at 22°C with 65% humidity and a 12 h light/dark cycle. After two days of feeding, they were injected subcutaneously with purified apoptin (0.03 mg/mouse) mixed with complete Freund’s adjuvant (Sigma-Aldrich, St. Louis, MO, USA) in a 1:1 ratio. The mice were subsequently injected three times with same quantity of protein mixed with incomplete Freund’s adjuvant (Sigma-Aldrich) at two-week intervals. At five days after the final injection, mouse blood was harvested using the eyeball blood sampling method, and pooled. The specificity of the antiserum was detected by western blotting, in which the prepared apoptin was considered the antigen, and the primary antibody the antiserum.

### Lentivirus construction and transduction of MSCs

Lentiviral particles carrying apoptin gene were prepared by transient co-transduction of pLV/Final-puro-EF1α-apoptin (Invitrogen Life Technologies) and a lentiviral packaging mix (Invitrogen Life Technologies) into 293FT cells (Invitrogen Life Technologies) using Lipofectamine 2000 (Invitrogen Life Technologies), according to manufacturer’s instructions. At 48 h after transfection, viral particles were harvested, filtered through a 0.45-*μ*m polyethersulfone membrane and concentrated by ultracentrifugation at 50,000 × g for 1 h at 4°C. Titers of concentrated lentivirus particles changed from 4×10^7^ to 9×10^7^ U/ml. Human MSCs were transduced with lentiviral particles at an infection multiplicity of 50. Following two rounds of infection, puromycin (HyClone Laboratories, Inc.) was added to the culture medium at a concentration of 1–5 *μ*g/ml and maintained for 2–3 days. The obtained MSC lines were defined as MSCs APOPTIN. At the same time, lentiviral particles carrying hrGFP were prepared with pLV/Final-puro-EF1α-hrGFP (Invitrogen Life Technologies) and transduced into MSCs in parallel to assess the modification efficiency of apoptin.

### Western blot analysis

MSCs APOPTIN or control cells were washed with cold PBS and immediately lysed in Laemmli buffer (Nanjing Keygen Biotech. Co. Ltd., Nanjing, China). Cell lysates were denatured at 100°C for 5 min and centrifuged at 10,000 × g for 5 min at 4°C. Supernatants were recovered, separated on 12% SDS-PAGE and transferred onto a 0.45-*μ*m polyvinylidene difluoride membrane (Millipore, Billerica, MA, USA). Following blocking the membrane with Tris-buffered saline-Tween-20 containing 5% non-fat milk (Mengniu Dairy, Inner Mongolia, China) for 1 h at room temperature, the membrane was incubated with the appropriate primary antibodies: Anti-apoptin polyclonal antiserum prepared in the present study (1:500) and monoclonal anti-GAPDH antibody (cat. no. ab8245; 1:10,000; Abcam, Cambridge, MA, USA), overnight at 4°C. The membrane was incubated with horseradish peroxidase-conjugated secondary antibody (cat. no. sc-2005; 1:10,000; Santa Cruz Biotechnology, Inc., Dallas, TX, USA) for 1 h at room temperature and the bands were detected using the SignalFire™ Enhanced Chemiluminescence reagent (Cell Signaling Technologies, Beverly, MA, USA) in a dark room.

### Co-culture experiments

A total of 1×10^5^ MSCs APOPTIN or MSCs were pre-plated in six-well plates overnight. Subsequently, three types of cells, including fibroblast hrGFP (1×10^5^), H460 hrGFP (4×10^5^) and H1299 hrGFP (4×10^5^), were added to these wells. After 48 h of culture, the viability of total cells was determined using a cell counting kit-8 (CCK-8; Dojindo Laboratories, Kumamoto, Japan) and analyzed as a percentage against control MSCs. The extent of apoptosis of cells with green fluorescence was recorded via microscopy (BX51; Olympus Corporation, Tokyo, Japan) following direct staining with DAPI (Sigma-Aldrich). At the same time, the presence of activated caspase-3 in H460 hrGFP was measured with an anti-active caspase-3 polyclonal antibody (Promega, Madison, WI, USA) at different time-points during co-culture. The media derived from 24 h incubation with MSCs APOPTIN and MSCs, were used to treat H460 cells for 48 h and the viability of the target cells was detected using the CCK-8 kit.

### Animal studies

Athymic nude mice (six weeks old) were purchased from Guangdong Medical Laboratory Animal Center (Foshan, China) and used in accordance with institutional guidelines under approved protocols. A total of 1×10^6^ H460 cells with or without 3×10^5^ MSCs or MSCs APOPTIN were suspended in 100 *μ*l PBS and subcutaneously injected into the flank area of nude mice. At the 14th day after the first injection, the same quantity of MSCs or MSCs APOPTIN were again injected into the same position. Subsequently, mice were examined three times a week and tumor sizes were calculated as previously described (10): volume = length × width^2^/2. On the 60th day, the tumor was excised after mice were sacrificed by cervical dislocation and the tumor mass was determined.

### Statistical analysis

Values are expressed as the mean ± standard deviation. The differences between experimental and control groups were analyzed using a two-tailed Student’s t-test employing SPSS version 12.0 (SPSS Inc., Chicago, IL, USA). P<0.05 was considered to indicate a statistically significant difference.

## Results

### Identification of anti-apoptin polyclonal antibody

**Figure 1:**
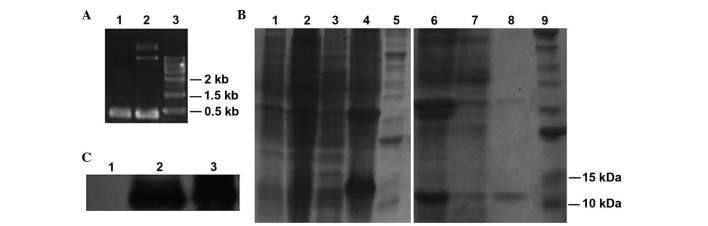
Preparation of anti-apoptin antibody and apoptin-modified MSCs. (A) Recombinant vector pET28b-apoptin was identified by PCR and double enzyme cutting. Lane 1, PCR product; lane 2, double enzyme cutting product. (B) Apoptin protein was induced and purified. Lane 1, total lysate of host BL21 (DE3) bearing pET28b; lane 2, total lysate of host BL21 (DE3) bearing pET28b-apoptin; lane 3, supernatant of BL21 (DE3) bearing pET28b-apoptin; lane 4, sediment of BL21 (DE3) bearing pET28b-apoptin; lane 5/9, protein marker; lane 6, sample used for Ni^+^ affinity chromatography; lane 7, eluate with PBS containing 100 mM imidazole; lane 8, eluate with PBS containing 200 mM imidazole. (C) The specificity of anti-apoptin antibody was confirmed by western blot analysis. Lane 1, negative control; lane 2, total lysate of host bacteria containg pET28b-apoptin; lane 3, purified apoptin. MSC, mesenchymal stem cell; PCR, polymerase chain reaction; PBS, phosphate-buffered saline.

To prepare the anti-apoptin polyclonal antibody, a prokaryotic expression vector pET28b-apoptin was constructed by double enzyme cutting and ligation reaction. As shown in Fig. 1A, the encoding fragment of apoptin was correctly inserted into the multiple cloning sites of pET28b. After pET28b-apoptin was transformed into host bacterial BL21 (DE3), the positive clone containing recombinant vector was induced to express apoptin with IPTG and low temperature. Subsequently, Ni^+^ ion affinity chromatography was used to purify the target protein. As shown in Fig. 1B, apoptin with a purity >90% was collected. The eluate containing apoptin was dialysed, concentrated and injected into BALB/c mice to prepare anti-apoptin polyclonal antibody. The western blotting results indicated that the polyclonal antibody specifically bound apoptin derived from the prokaryotic expression system (Fig. 1C).

### Generation, identification and characterization of apoptin-modified MSCs

**Figure 2:**
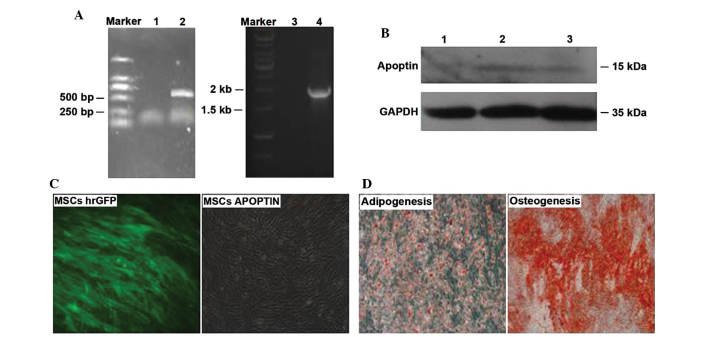
Construction of apoptin-modified MSCs. (A) Lentiviral vector pLV/Final-puro-EF1α-apoptin was identified by PCR. Lane 1/3, negative control; lane 2, amplification product of fragment of apoptin; lane 4, amplification product of fragment of EF1α promoter. (B) Expression of apoptin was confirmed by western blot analysis. Lane 1, negative control; lane 2, MSCs APOPTIN 1; lane 3, MSCs APOPTIN 2. (C) Morphology of MSCs hrGFP and MSCs APOPTIN. (D) Adipogenesis and osteogenesis differentiation of MSCs APOPTIN. Magnification, ×200. MSCs APOPTIN, mesenchymal stem cells expressing apoptin; PCR, polymerase chain reaction; hrGFP, humanized Renilla green fluorescence protein.

Firstly, the correction of lentiviral expression vector pLV/Final-puro-EF1α-apoptin was confirmed by PCR (Fig. 2A) and sequencing (data not shown). In addition, western blotting also revealed that apoptin protein was expressed in MSCs APOPIN and not in control MSCs (Fig. 2B). Lentiviral particles containing apoptin or hrGFP gene were prepared in 293FT cells. Subsequently, MSCs were transfected with the lentiviral particles. Following puromycin selection, >90% of hrGFP-modified MSCs expressed hrGFP (Fig. 2C). Due to the same experimental conditions, it was hypothesized that the modification efficiency in MSCs APOPTIN was similar. Furthermore, it was demonstrated that MSCs APOPTIN may be induced into adipocytes or osteoblasts *in vitro* under the appropriate conditions (Fig. 2D).

### MSCs APOPTIN induce lung tumor cell apoptosis by activating caspase-3 within target cells

**Figure 3:**
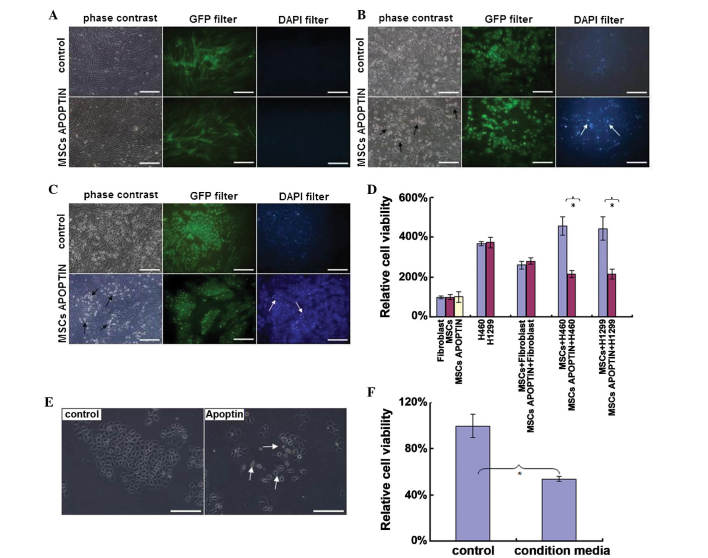
MSCs APOPTIN selectively induce apoptosis in lung cancer cells, but not in normal cells. MSCs APOPTIN were co-cultured with (A) human fibroblast hrGFP, (B) H460 hrGFP and (C) H1299 hrGFP for 48 h. The mixed cells were observed using phase-contrast microscopy with detection of apoptotic/necrotic cells via GFP and DAPI, respectively (scale bar, 200 *μ*m). (D) Cell viability in a co-culture system was measured using the CCK-8 kit and expressed as the percentage of control MSCs. ^*^P<0.01. Results are representative of three independent experiments. (E) Morphology of H460 cells treated with media derived from MSCs APOPTIN and MSCs. (F) Effect of different media on H460 cells as in E was evaluated using the CCK-8 kit. Condition media represents media the MSCs APOPTIN were grown in for 12 h. ^*^P<0.01. Results are representative of three separate experiments. (A-C and E) Scale bars represent 200 *μ*m. (B, C and E) Arrows indicate apoptotic and dead cells. hrGFP, humanized Renilla green fluorescence protein; MSCs APOPTIN, mesenchymal stem cells expressing apoptin; CCK-8, cell counting kit-8.

As shown in Fig. 3A–D, MSCs APOPTIN induced apoptosis in the lung cancer cell lines H460 and H1299, as represented by detachment of adherent GFP-positive cells and the appearance of cellular debris in DAPI-GFP double positive cells, but not in fibroblast cells. The percentage of viable cells detected using the CCK-8 kit also indicated that MSCs APOPTIN inhibited the proliferation of transformed cells (P<0.05). In addition, control MSCs had no detectable effect on these cells, whereas media derived from MSCs APOPTIN inhibited H460 cell proliferation (Fig. 3E and F).

**Figure 4:**
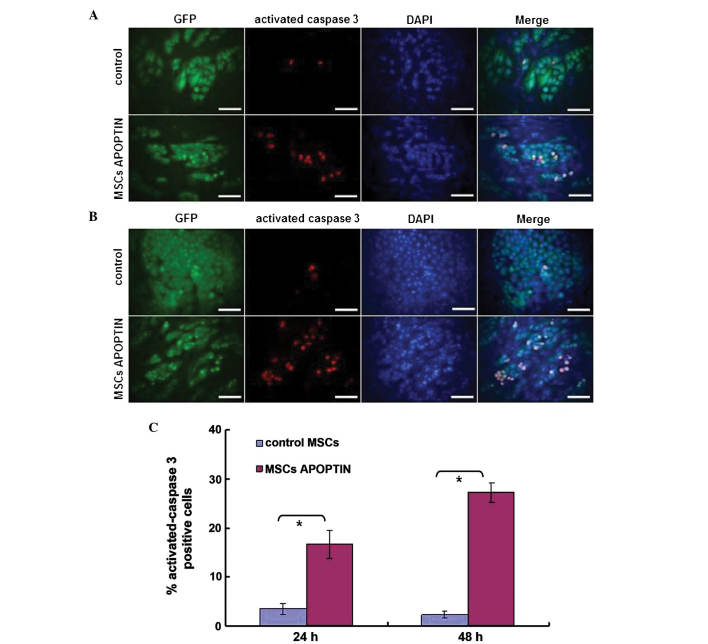
MSCs APOPTIN induce tumor cell apoptosis by activating caspase-3 within target cells. H460 hrGFP were co-cultured with MSCs APOPTIN for (A) 24 h and (B) 48 h, respectively. Activated caspase-3 within H460 hrGFP were detected by immunofluorescence staining. Scale bars represent 100 *μ*m. (C) Percentage of activated caspase3-positive H460 hrGFP was quantified using ImagePro 5.0. ^*^P<0.01. Results are representative of three separate experiments. hrGFP, humanized Renilla green fluorescence protein; MSCs APOPTIN, mesenchymal stem cells expressing apoptin.

To further examine the mechanism of action of apoptin, the activation of caspase-3 within target cells was measured. As shown in Fig. 4, the percentage of H460 cells positive for activated caspase-3 in the MSCs APOPTIN group was markedly higher than that in the control MSCs group (16.5±2.9% at 24 h and 27.3±2.0% at 48 h vs. 3.4±1.1% at 24 h and 2.2±0.6% at 48 h). It was observed that the percentage of target cells positive for activated caspase-3 evidently increased with prolongation of co-culture time in the MSCs APOPTIN group, whereas there was no marked change in the control group. From these results, it was concluded that apoptin derived from MSCs APOPTIN effectively induced tumor cell apoptosis via activation of caspase-3 within target cells.

### MSCs APOPTIN reduces subcutaneous lung tumor growth in vivo

**Figure 5:**
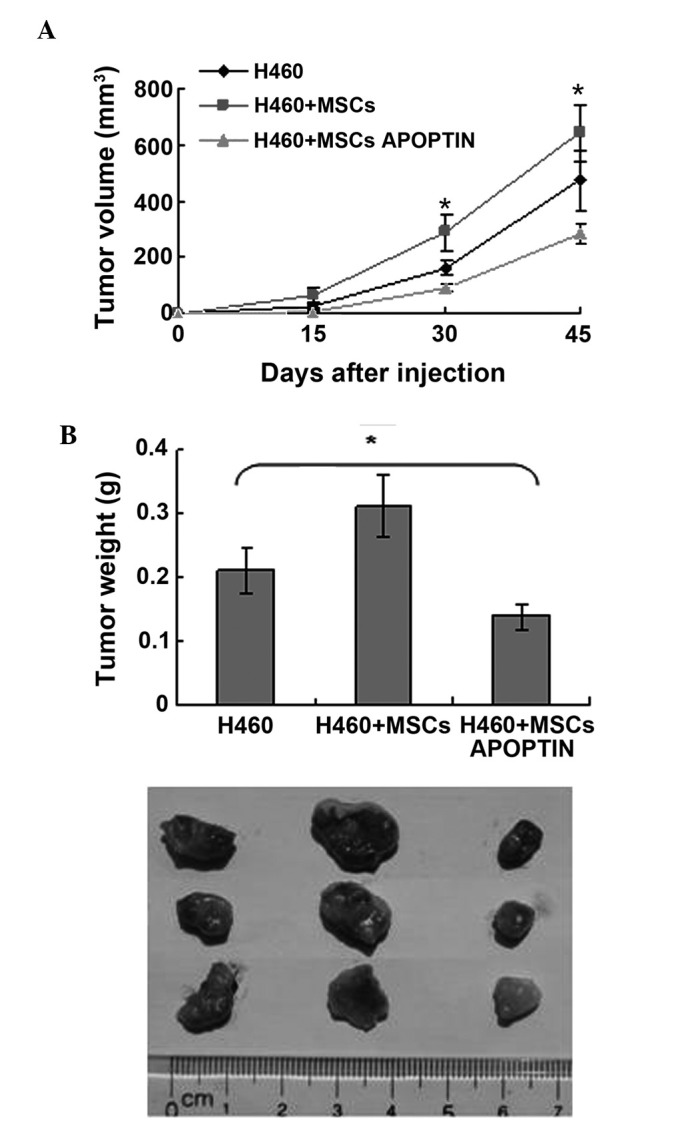
MSCs APOPTIN inhibit tumor cell growth in nude mice. (A) Tumor sizes were dynamically monitored after H460 cells with or without MSCs or MSCs APOPTIN were injected into nude mice (n=3 mice/group, three separate experiments). ^*^P<0.05, H460 group vs. H460+MSCs APOPTIN group. (B) Tumor mass weight and morphology were compared at the end of incubation *in vivo*. ^*^P<0.05, H460 group vs. H460+MSCs APOPTIN group. MSCs APOPTIN, mesenchymal stem cells expressing apoptin.

To further confirm the *in vitro* finding of MSCs APOPTIN *in vivo*, a xenotransplantation model of lung carcinoma was established and the kinetics of tumor growth were assessed. As shown in Fig. 5, tumor masses began to appear at ~23 days following inoculation in the single H460 group. MSCs APOPTIN delayed the appearance and inhibited the growth of the tumor mass. The average weight of tumor mass in the H460+MSCs APOPTIN group, the H460 group, and the H460+MSCs group was 0.14±0.02, 0.21±0.04 and 0.31±0.05 g, respectively. By contrast, MSCs accelerated the growth of the tumor mass in the early phase of subcutaneous tumor growth (up to 50 days). However, the difference in tumor size and weight between the tumor cell group containing MSCs and tumor cell group alone was eliminated at approximately 60 days.

## Discussion

MSCs have the advantage of being an optimal cell delivery vehicle for gene therapy (17). As a small molecular protein, apoptin has been used to suppress tumor cell growth (18). However, the limitations of the protein drug, such as the short half life, indicated a requirement for a novel method of its delivery (15). Therefore, it was assessed whether apoptin-modified MSCs may inhibit tumor growth.

Initially, a specific anti-apoptin antibody was prepared by immunizing mice with purified apoptin protein derived from a prokaryotic expression system.

It is important to maximally improve the modification efficiency of MSCs in order to realize the expected goal. Compared with other gene modification systems, including adenoviral or retroviral vectors, the human immunodeficiency virus-based lentivirus system may stably transduct cells at a different mitotic stage, which is very applicable to MSCs, which are often quiescent (19,20). Several studies have reported that lentiviral vectors may effectively deliver target genes into MSCs (19,21). Correspondingly, the present results also indicated that the apoptin gene may be stably integrated into MSCs via a lentiviral vector and expressed by host cells. In addition, the differentiation capacity of MSCs was not affected by apoptin or the lentiviral vector.

A further problem which requires development is to guarantee that apoptin enters target cells following its expression by MSCs. Although the detailed mechanism of apoptin-induced apoptosis remains to be elucidated, it is certain that apoptin-induced apoptosis depends on the entering of apoptin into target cells (22). As apoptin itself has no associated signal sequences, one secreting signal and one PTD were introduced into the N-terminus of apoptin during construction of the lentiviral expression vector according to previous studies (23,24). The two signal sequences may assist apoptin to be secreted from host cells and then transferred into target cells. As demonstrated by the present results, apoptin may be successfully secreted into the extracellular space and enter target cells.

According to associated studies, >70 human tumor cell lines are susceptible to the pro-apoptotic effect of apoptin (11,12). From these studies, it was found that apoptin exerts its apoptosis-inducing function mainly through direct expression in tumor cells or in the form of a fusion protein. It is likely that these modes may narrow its clinical application. The present results indicated that apoptin-modified MSCs may induce apoptosis in lung cancer cell lines, while sparing normal cells. It is evident that the combination of MSCs and apoptin may broaden their application. Previous studies have indicated that cell apoptosis induced by apoptin is associated with the activation of typical caspases (25,26). This theory was further confirmed by the present results, which demonstrated that apoptin-modified MSCs markedly activated caspase-3 within target cells.

Of note, apoptin-modified MSCs may decrease the growth of tumors in a xenograft mouse model, although native MSCs promoted their growth in comparison with tumor cells alone, which is in agreement with the results of a previous study (27). The possible explanation may be that the pro-apoptotic capacity of apoptin overcomes the tumor-supportive capacity of MSCs, which eventually results in the inhibition of tumor growth by apoptin-modified MSCs.

In conclusion, the present study indicated that MSCs may be effectively modified by insertion of the apoptin gene via a lentiviral transduction system. The apoptin-engineered MSCs may inhibit tumor cell growth *in vitro* and *in vivo*. Thus, apoptin-modified MSCs may be a novel option in cancer therapy.
